# The mannosylated extracellular domain of Her2/neu produced in *P. pastoris *induces protective antitumor immunity

**DOI:** 10.1186/1471-2407-9-386

**Published:** 2009-10-30

**Authors:** Alexios Dimitriadis, Chrysanthi Gontinou, Constantin N Baxevanis, Avgi Mamalaki

**Affiliations:** 1 Laboratory of Molecular Biology and Immunobiotechnology, Department of Biochemistry, Hellenic Pasteur Institute, 127 Vas. Sofias Ave., 11521 Athens, Greece; 2 Cancer Immunology and Immunotherapy Center, Saint Savas Cancer Hospital, 171 Alexandras Ave., 11522 Athens, Greece

## Abstract

**Background:**

Her2/neu is overexpressed in various human cancers of epithelial origin and is associated with increased metastatic potential and poor prognosis. Several attempts have been made using the extracellular domain of Her2/neu (ECD/Her2) as a prophylactic vaccine in mice with no success in tumor prevention.

**Methods:**

The extracellular domain of Her2/neu (ECD/Her2) was expressed in yeast *P. pastoris*, in a soluble highly mannosylated form. The immune response of the immunization with this recombinant ECD/Her2 was analyzed using immunoprecipitation and western blot analysis, proliferation and cytotoxicity assays as well as specific tumor growth assays.

**Results:**

Mannosylated ECD/Her2 elicited a humoral response with HER2/neu specific antibodies in vaccinated mice, which were able to reduce the proliferation rate of cancer cells *in vitro*. Moreover, it elicited a cellular response with Her2/neu-specific CTL capable of lysing tumor cells, *in vitro*. When immunized Balb/c and HHD mice were challenged with Her2/neu-overexpressing cells, tumor growth was inhibited.

**Conclusion:**

Here we report on the efficacy of the extracellular domain of human Her2/neu produced in yeast *P. pastoris*, which confers mannosylation of the protein, to act as a potent anti-tumor vaccine against Her2/neu overexpressing tumors. Specific cellular and humoral responses were observed as well as efficacy.

## Background

The Her2/neu (ErbB2) gene encodes a 185 kDa transmembrane glycoprotein that belongs to the family of epidermal growth factor receptors. It consists of a 620 aa extracellular domain, followed by a 23 aa transmembrane domain and a 490 aa intracellular domain with a tyrosine kinase activity [1]. It is a ligand-less receptor and it is the preferred heterodimerization partner for ligand-bound EGFR, Her3 and Her4, serving as a co-receptor. Any alteration of the firmly regulated EGF receptor signaling pathways leads to cellular abnormalities and tumorigenesis [2].

The Her2/neu gene has a low expression in normal tissues. However, it is amplified and overexpressed in ~30% of invasive breast carcinomas and it is associated with increased metastatic potential and poor prognosis. Overexpression of the Her2/neu receptor is also observed in various other human cancers, including lung, ovary, kidney, and bladder [3]. Several reports have shown that the Her2/neu molecule is immunogenic, since it may generate antibodies and peptide-specific CTL response in some patients [4]. Therefore Her2/neu is an attractive target for active immunotherapy. DNA- and peptide-based vaccines were able to break tolerance and generate tumor antigen-specific immunity in animal models [5-8]. However, when the extracellular domain of the Her2/neu protein, produced in cell lines which confer normal mammalian glycosylation, was used as a vaccine, it could not confer protection against Her2/neu overexpressing tumors [9-11]. An effective immune response was elicited only when ECD/Her2 was fused to cytokines [12] or combined with antibodies fused to cytokines [11]. Given that antigen presenting cells (APCs) do recognize and direct mannosylated antigens for degradation [13], it was recently shown that the use of fungal systems to mannosylate vaccine candidates can enhance immunogenicity in the context of CD4^+ ^[14] and CD8^+ ^T cells [15]. The present study utilizes the mannosylated extracellular domain of the human HER2/*neu *receptor (ECD/Her2) produced in yeast *P. pastoris *to reduce tumor growth. ECD/Her2 elicited a humoral response in vaccinated mice with specific antibodies against Her2/neu that were able to reduce the proliferation rate of cancer cells *in vitro*. It, also, elicited a cellular response with Her2/neu-specific CTL capable of lysing tumor cells *in vitro*. When Balb/c and HHD vaccinated mice were challenged with Her2/neu overexpressing cells, tumor growth was inhibited. These results suggest that ECD/Her2 is a good candidate for a tumor antigen vaccine as it prolongs tumor free survival and overall survival of vaccinated mice.

## Methods

### Animals

Female BALB/c obtained from Harlan Laboratories (Indianapolis, IN, USA) and HHD mice (β_2 m_^-/-^, H-2D^b-/- ^and expressing a HLA.A2.1 monochain composed of a chimeric heavy chain, α1 and α2 domains of HLA-A*0201 and the α3 intracellular domain of D^b ^[16]) obtained from Institut Pasteur (Paris, France), were used.

All mice were maintained in pathogen-free conditions in the animal facilities of the Hellenic Pasteur Institute. Experiments were performed according to the Greek and European regulations on Animal Welfare and with Public Health Service recommendations.

### Cell lines

D2F2/E2, a mouse mammary tumor cell line stably transfected with the human Her2/neu molecule, was previously described [17]. This cell line, as well as the parental D2F2 cell line, was maintained in hi-glucose DMEM, supplemented with 100 units/mL Penicillin, 100 μg/mL Streptomycin, 10% FBS, 10% NCTC 109, 1% non-essential amino acids and 5% Sodium Bicarbonate.

ALC.A2.1.hHer2, a murine lymphoma cell line stably transfected with the HLA.A2.1 and the human Her2/neu molecule [8] and the SK-BR-3 cell line were maintained in RPMI 1640, supplemented with 100 units/mL Penicillin, 100 μg/mL Streptomycin and 10% FBS.

All cell lines were maintained at 37°C under a 5% CO_2 _- 95% air atmosphere.

### Soluble expression of ECD/Her2 in yeast Pichia pastoris

The extracellular domain of human Her2/neu receptor (ECD/Her2, aminoacid residues 1 - 627) was previously enzymatically amplified by PCR and subcloned into the expression vector pPICZaC (Invitrogen, Carlsbad, California, USA) for soluble expression in yeast *P. pastoris *[18]. Protein production was induced with methanol while cells were grown at 30°C for 72 h. The culture supernatant was passed through a 0.22 - μm membrane filter, concentrated using the Minitan Ultrafiltration System (Millipore, Billerica, Massachusetts, USA) equipped with 30 kDa cut-off membrane and then dialyzed extensively against 20 mM sodium phosphate buffer pH 7.4, containing 0.5 M NaCl and 10 mM Imidazole. The concentrated protein was purified over a Ni^2+ ^affinity chromatography using HisTrap FF column and a FPLC AKTA Purifier system (Amersham Biosciences, Munich, Germany) under native conditions, according to the manufacturer's protocol. The recombinant protein was filtrated and stored at -80°C.

In order to deglycosylate the produced molecule, approximately 3 μg of ECD/Her2 were denatured at 100°C for 10 min in a buffer containing 0.5% SDS and 1% β-mercaptoethanol. 50 mM sodium phosphate and natural phosphate (NP) to a final concentration of 1% were added, and the protein was incubated with 500 U N-glycosidase F (PNGase F, New England Biolabs, Ipswich, Massachusetts, USA) for 1 h at 37°C, in a total reaction volume of 80 μl.

### Immunization protocol

Mice at the age of 6-8 weeks were immunized (day 0) by subcutaneous (s.c.) injection at the base of the tail with either PBS (control group) or 25 μg ECD/Her2 emulsified in complete Freund's adjuvant (CFA) (SIGMA, St. Louis, Missouri, USA). Two boosts of the same dose emulsified in incomplete Freund's adjuvant (IFA) (SIGMA) were carried out 21 and 42 days later. Mice weight was measured as an indicator of toxicity caused by vaccinations. Blood samples were taken by tail bleeding before each vaccination, as well as 10, 21 and 42 days after the last vaccination.

### Tumor model

10 days after the last immunization mice were challenged by s.c. injection into the left flank of 1 × 10^5 ^D2F2/E2 or D2F2 tumor cells (BALB/c mice), or 3 × 10^5 ^ALC.A2.1.hHer2 tumor cells (HHD mice). Tumor cells were suspended in sterile PBS. Animals were monitored twice per week for the development of palpable tumors. Tumors were measured by calliper and the tumor volume was calculated using the formula tumor volume = (length × width^2^)/2. Mice were sacrificed with euthanasia when the tumor volume grew up to 2000 mm^3^.

### ELISA assay

Sera from immunized mice were analyzed for anti-ECD/Her2 reactivity in enzyme-linked immunosorbent assays (ELISA). Ninety-six-well microtiter plates were coated with 20 μg/mL ECD/Her2 for 16 hrs at 4°C. Non-specific binding sites were blocked with PBS/3% BSA for 1 h at 37°C. Plates were incubated with sera dilutions in PBS/1% BSA, in triplicates, overnight at 4°C. Anti-ECD/Her2 antibodies were detected using goat anti-mouse IgG conjugated to horseradish peroxidase (DAKO Cytomation, Glostrup, Denmark) in PBS/1% BSA, for 1 h at 37°C. Reactions were developed after the addition of TMB substrate, and then stopped with the addition of 50 μL/well 1 M sulfuric acid. Absorbance was read on a microtiter plate reader (BioRad, Hercules, California, USA).

### Immunoprecipitation and Western blot analysis

Pooled sera from groups of immunized mice were used to immunoprecipitate human Her2/neu from SK-BR-3 cell membrane lysates. 1 μg Herceptin (Trastuzumab) was used as a positive control, and PBS as negative control. 200 μL beads Gammabind Sepharose (Amersham, Munich, Germany) were pre-incubated with SK-BR-3 cell membrane lysate for 2 hours at room temperature, in order to avoid any non-specific binding. 2 μL of sera were incubated with 600 μg of cell membrane lysate overnight at 4°C. 30 μL of Gammabind Sepharose were added and the solution was gently rocked for 2 h at room temperature. Sepharose pellets were washed twice with TSA solution (0.01 M Tris-HCl pH 8, 0.14 M NaCl, 0.025% NaN_3_) containing 0.1% Triton-X and once with TSA alone.

Immunoprecipitates were separated on 8% SDS-PAGE gels and blotted onto PROTRAN nitrocellulose membranes (WHATMAN, Dassel, Germany). The membranes were blocked with 5% non-fat milk in PBS, 0.1% Tween-20. Her2/neu was detected by immunoblotting with C-18 rabbit anti-Her2/neu (Santa Cruz Biotechnology, Santa Cruz, California, USA), overnight at 4°C. Goat anti-rabbit IgG conjugated to horseradish peroxidase (DAKO Cytomation, Glostrup, Denmark) was the secondary antibody and bound antibodies were visualized by chemiluminescence using the ECL-Western Blot detection system (Amersham, Munich, Germany). Membranes were exposed to X-ray film (Amersham, Munich, Germany).

### SK-BR-3 in vitro proliferation assay

Single cell suspensions of 5 × 10^3 ^SK-BR-3 cells were left to adhere overnight at 96-well culture plates at 100 μL/well of RPMI-1640 containing 5% FBS. Pooled sera from each group of vaccinated mice were depleted of complement by incubation at 56°C for 30 minutes, diluted in serum-free culture medium to give a final working dilution of 1:100 and added to SK-BR-3 cells, which were then incubated for 3 days at 37°C. The number of living cells was determined by CellTiter 96 Aqueous One Solution Cell Proliferation Assay (Promega, Madison, Wisconsin, USA).

### Cytotoxicity assay

ECD/Her2 or PBS immunized mice were used 10 days after the last vaccination, to isolate CD8^+ ^T cells from total immune splenocytes by negative selection using the CD8a^+ ^T Cell Isolation Kit (Miltenyi Biotec, Bergisch Gladbach, Germany). Freshly isolated CD8^+ ^T cells were used for cytotoxicity assays using the CytoTox 96 Non-Radioactive Cytotoxicity Assay (Promega, Madison, Wisconsin, USA). Briefly, 5000 D2F2 or D2F2/E2 cells were coated in each well of a 96-well v-bottom culture plate as target cells. As effector cells, CD8^+ ^T cells from immunized mice were used at the indicated E:T ratios. Cytotoxicity is measured upon the lactate dehydrogenase (LDH) released upon cell lysis. The percentage of cytotoxicity is calculated according to the formula % cytotoxicity = 100 × (experimental value - effector spontaneous LDH release - target spontaneous LDH release)/(target maximum LDH release - target spontaneous LDH release). Cytotoxicity values were considered to indicate significant lytic activity of a target when the differences between mean values for % lysis of target cells from ECD/Her2 vaccinated mice and PBS vaccinated mice were >10% and statistically significant (*p *< 0.05).

### Statistical analysis

The statistical significance of differential findings between experimental groups was determined by student's *t *test. Findings were regarded as significant, if *p *values were < 0.05. Tumor sizes among the groups were compared with Mann-Whitney U test using the SPSS (v.16.0) program. Kaplan-Meier curves were plotted for tumor free analysis. All *p *values were two tailed and considered significant when *p *< 0.05.

## Results

### Soluble expression of ECD/Her2 in yeast *P. pastoris*

**Figure 1:**
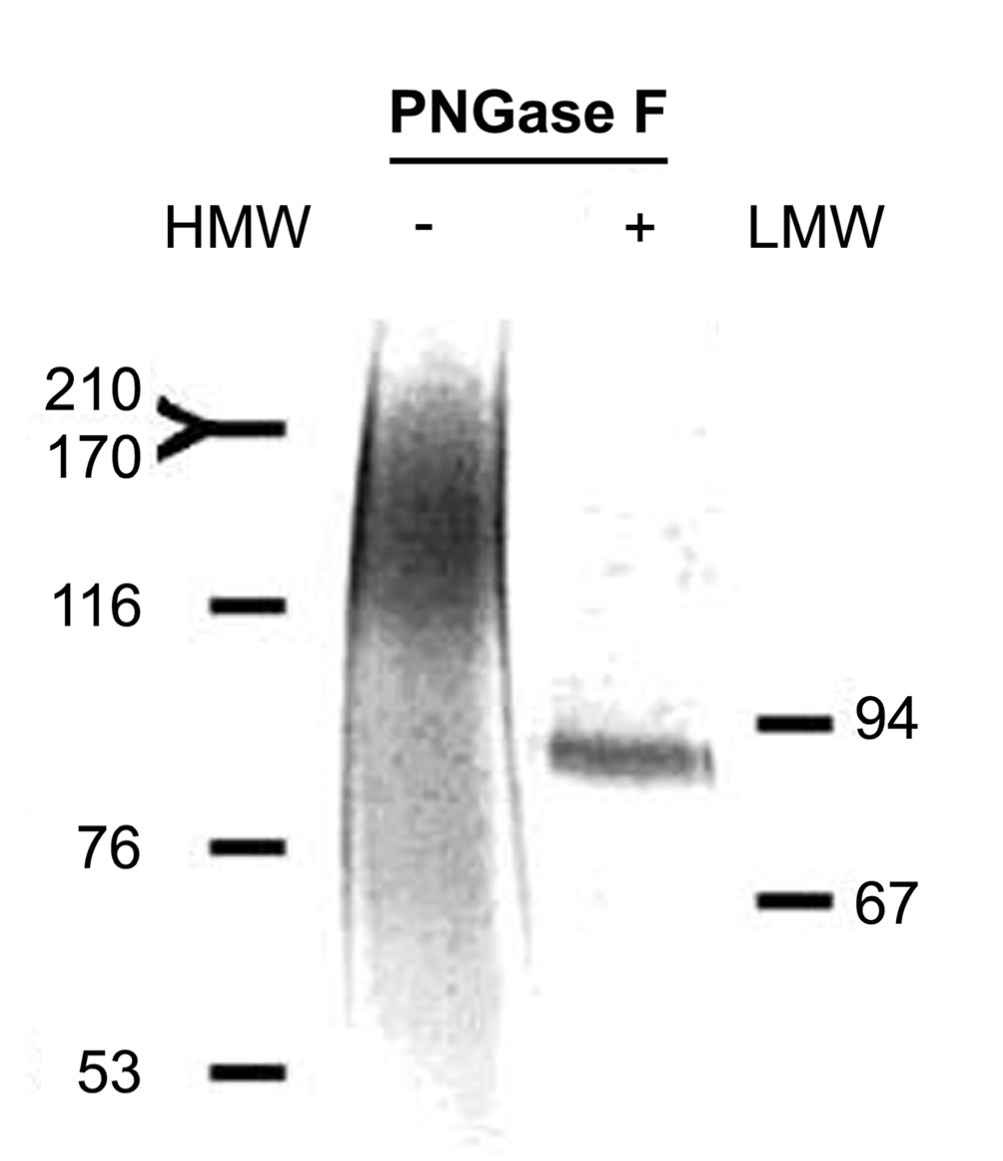
**Deglycosylation of ECD by peptide N-glycosidase F (PNGase F)**. Purified ECD incubated for 1 h at 37°C with (+) or without (-) PNGase F was analyzed in 8% SDS-PAGE. HMW, LMW: high and low molecular weight markers.

The recombinant human ECD/Her2 expressed in soluble form in yeast *P. pastoris*, was purified using Ni^2+ ^affinity chromatography. The molecular size of the product was estimated as ~120-210 kDa higher than that predicted from the amino acid sequence (73 kDa). This difference was shown to be due to glycosylation of the molecule since enzymatic deglycosylation using peptide *N*-glycosidase F resulted in a reduction in the apparent molecular size to about 85 kDa (figure 1). These results show that the ECD/Her2 is highly mannosylated, as in *P.pastoris *the N-linked and O-linked glucans are terminally mannosylated. The yield of the isolated protein was approximately 0.4 mg/l.

### Vaccination with the mannosylated ECD/Her2 expressed in *P. pastoris *elicits a humoral response against Her2/neu

**Figure 2:**
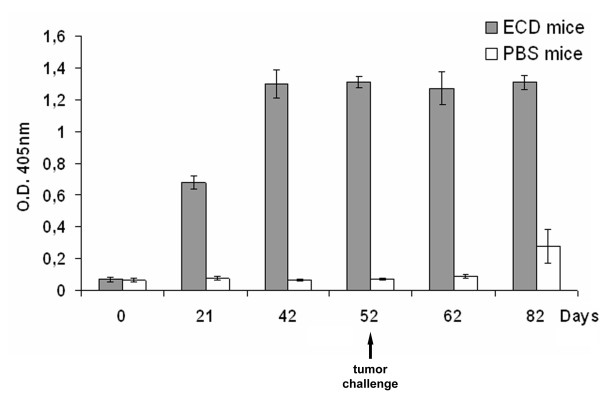
**Vaccination with the mannosylated ECD/Her2 expressed in *P. pastoris *elicits a humoral response**. Anti-ECD/Her2 antibodies in the sera of mice immunized with ECD/Her2 or PBS, as measured with enzyme-linked immunosorbent assay (ELISA). Sera were collected at various times. Pooled sera from the two groups of mice were assayed at a 1:1000 dilution. Columns and error bars represent the means and the range of values obtained from 3 independent experiments. ECD mice: ECD/Her2 vaccinated mice. PBS mice: PBS vaccinated mice (control group).

To determine whether the recombinant mannosylated ECD/Her2 expressed in yeast *P. pastoris *could induce a humoral immune response, groups of BALB/c mice were immunized with the recombinant protein or PBS emulsified in CFA s.c., followed by two boosts in IFA on days 21 and 42. Immunized mice with the recombinant human ECD/Her2 developed specific antibodies against ECD/Her2 as measured by ELISA (figure 2). The levels of anti-ECD/Her2 antibodies detected after the first immunization (day 21) were increased by two times after the first boost (day 42) reaching a plateau. The antibody titer remained high (above 1:10^5^) even 40 days after the last vaccination (day 82). Anti-ECD/Her2 antibodies were not present in control mice which were injected with PBS, while the increased background level 30 days after the tumor challenge (day 82-see below) is due to the Her2/neu molecule that is expressed in the surface of D2F2/E2 transfected cell lines. Mice weight was measured as an indicator of toxicity caused by vaccinations and no differences were obtained compared to PBS vaccinated mice (data not shown).

**Figure 3:**
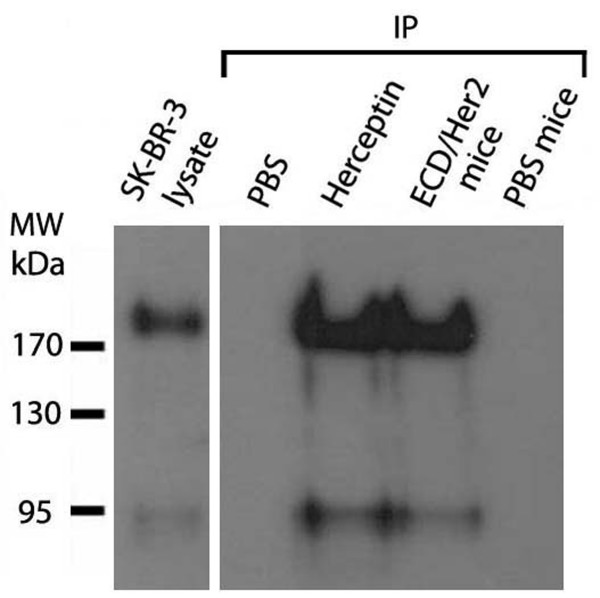
**Specificity of antibodies against native Her2/neu**. Antibodies from sera of ECD/Her2-immunised mice are specific against the human Her2/neu molecule, as seen with immunoprecipitation assay. Her2/neu protein extracted from SKBR- cells immunoprecipitated with antibodies from immunized sera and then subjected to immunoblotting using C18 anti-Her2/neu antibody. Sera ECD mice: Sera from ECD/Her2 vaccinated mice. Sera PBS mice: Sera from PBS vaccinated mice (control group). Positive control: Herceptin. Negative control: PBS. SK-BR-3 lysate: SK-BR-3 cell lysate was used for detection of Her2/neu molecular weight.

In order to investigate whether these antibodies produced in ECD/Her2- immunised mice were able to react with the native human Her2/neu, we performed immunoprecipitation assay. As shown in figure 3, the antibodies produced in ECD/Her2-immunized mice were specific against the native Her2/neu receptor of SK-BR-3 cells. These antibodies were not present in sera from PBS-immunised mice.

### In vivo antitumor activity of the mannosylated ECD/Her2 in BALB/c mice

**Figure 4:**
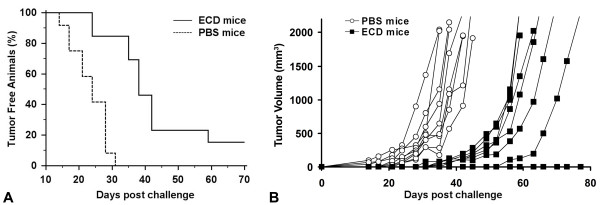
**Immunization with ECD/Her2 induces strong antitumor immunity in mice**. Groups of 9 mice were injected as described in Materials and Methods either with ECD/Her2 or with PBS on days 0, 21, and 42. 10 days after the last injection, mice were inoculated s.c. with 10^5 ^D2F2/E2 cells. This is one representative experiment of 3 performed. **A**. Kaplan-Meier curve was plotted for tumor free analysis. Unbroken line represents ECD/Her2 vaccinated mice and broken line PBS vaccinated mice (control group). **B**. Tumor growth in groups of vaccinated mice. The volume of individual tumors was calculated as (length × width^2^)/2. Curves represent individual mice. Black square represents ECD/Her2 vaccinated mice and open circle PBS vaccinated mice (control group). *p *< 0.01.

The *in vivo *antitumor activity of the recombinant mannosylated ECD/Her2 was tested in BALB/c mice. Groups of 9 mice were injected s.c. with the recombinant ECD/Her2 or PBS on day 0, followed by two boosts on days 21 and 42. 10 days after the last boost (day 52), 1 × 10^5 ^human Her2/neu expressing syngeneic murine cells D2F2/E2 or wild-type D2F2 were injected s.c. into the left flank of vaccinated mice. Tumors grew progressively in all PBS and ECD/Her2 immunised mice injected with D2F2 cells (data not shown). However, as shown in figure 4A, ECD/Her2 immunization significantly prolonged tumor free survival in D2F2/E2 injected mice (day 58) compared to PBS immunized mice (day 31), (*p *< 0.01). 2 mice became long-term survivors while the rest survived up to day 72 versus day 52 in the PBS-immunised mice (*p *< 0.01) (figure 4B). These data suggested that the vaccination of BALB/c mice with the mannosylated ECD/Her2 delayed the onset of tumor growth and in 2 mice provided full protection.

### Immune sera inhibits the growth of SK-BR-3 in vitro

**Figure 5:**
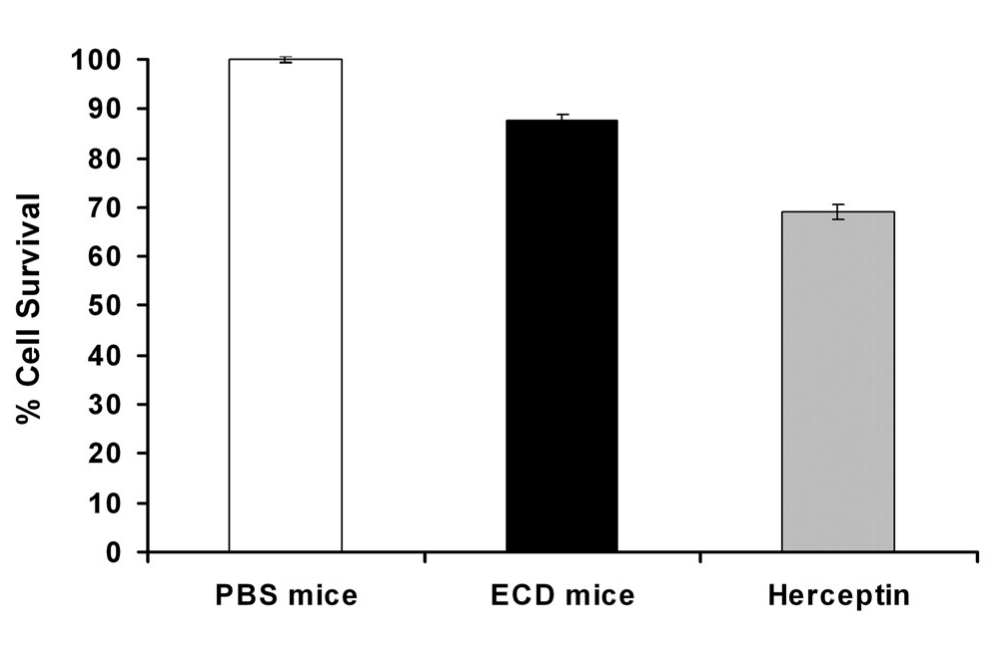
**Influence of immunized sera on the *in vitro *proliferation of SK-BR-3 cells**. Diluted sera from ECD/Her2-immunized mice were able to reduce the proliferation rate by 13% (*p *< 0.05). SK-BR-3 cells were incubated with complement inactivated pooled immune sera obtained from vaccinated mice one day prior to cell inoculation. Immune sera were diluted 1:100. Columns and error bars represent the means and the range of values obtained from 3 independent experiments. Positive control: Herceptin.

We next exploited the anti-proliferative effect of antibodies produced in ECD/Her2-immunized mice on the breast cancer cell line SK-BR-3 which overexpress Her2/neu. Diluted 1:100 pooled sera from vaccinated mice were added to the cells and cell proliferation was determined by MTS assay. Figure 5 shows that the diluted sera from ECD/Her2-immunized mice were able to reduce the proliferation rate of SK-BR-3 by 13% (*p *< 0.05). In contrast, no cell growth arrest was noted with sera from PBS-vaccinated mice, whereas Herceptin that was used as positive control antibody showed a strong inhibition effect (35%) at 6 nM. These data show that the antibodies contained in sera from ECD/Her2-immunized mice have significant anti-proliferative effects in SK-BR-3 cells.

### CD8^+ ^T cells from immunized mice are able to lyse tumor target cells

**Figure 6:**
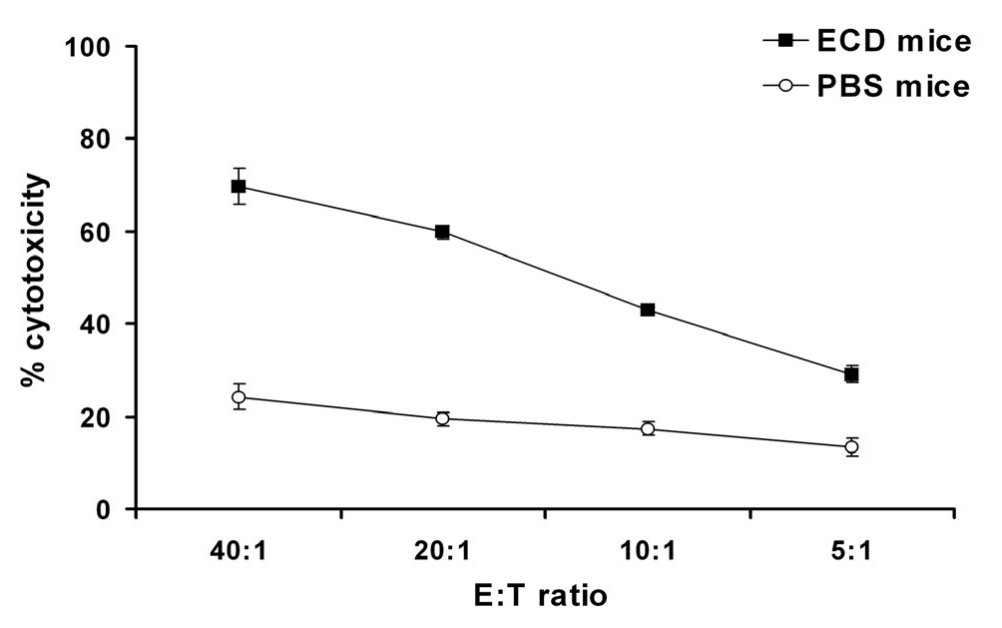
**CD8^+ ^T cells from immunized mice are able to lyse tumor target cells**. *In vitro *CTL activity of CD8 T lymphocytes isolated from spleens of BALB/c mice immunized with ECD/Her2 (black square) or PBS (open circle). CD8 T cells from these mice were used as CTL effectors against D2F2/E2 targets at the indicated E/T ratios and exhibited significantly increased lytic activity (*p *< 0,01). Points and error bars represent the means and the range of values obtained from 3 independent experiments.

Having demonstrated that ECD/Her2 vaccination elicits antibodies capable of reducing the proliferation rate of Her2-overexpressing tumor cells, we then sought to evaluate Her2/neu-specific CTL responses induced in the same mice. Splenocytes were prepared 10 days after the last immunization, and CD8^+ ^T cells were isolated using CD8a^+ ^T Cell Isolation Kit. As shown in figure 6, CD8^+ ^T cells isolated from PBS-immunized mice showed basal lytic activity against D2F2/E2 cells, while CD8^+ ^T cells isolated from ECD/Her2-immunized mice exhibited significantly increased lytic activity (*p *< 0.01). Negligible levels of cytotoxicity against wild type D2F2 cells were observed with CD8^+ ^T cells isolated from ECD/Her2- or PBS-immunized mice (data not shown).

### In vivo antitumor activity of the recombinant ECD/Her2 in HHD mice

**Figure 7:**
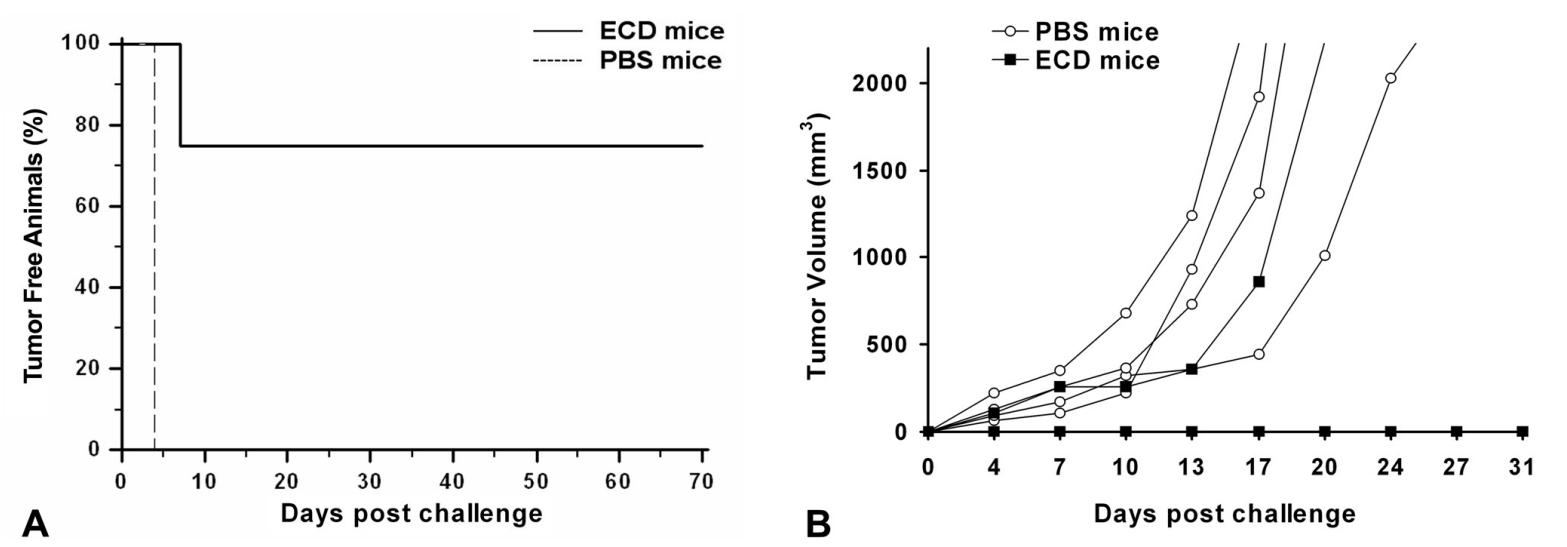
**Protective effect induced by immunization with ECD/Her2 in HHD mice**. **A**. Kaplan-Meier curve was plotted for tumor free analysis. Unbroken line represents ECD/Her2 vaccinated mice and broken line PBS vaccinated mice (control group). **B**. Tumor growth in groups of vaccinated mice. The volume of individual tumors was calculated as (length × width^2^)/2. Curves represent individual mice. Black square represents ECD/Her2 vaccinated mice and open circle PBS vaccinated mice (control group).

The *in vivo *antitumor activity of the recombinant ECD/Her2 was also evaluated in a second animal model. Groups of HHD mice were injected s.c. with ECD/Her2 (n = 4) or PBS (n = 4) on day 0, followed by two boosts on days 21 and 42. Specific antibodies against the ECD/Her2 and the native Her2/neu were developed in ECD/Her2 vaccinated mice that were able to reduce the proliferation rate of SK-BR-3 cells (data not shown). 10 days after the last boost (day 52) mice were challenged s.c. into the left flank with 3 × 10^5 ^ALC tumor cells stably transfected with Her2/neu and HLA.A2.1 (ALC.A2.1.hHer2). Tumors grew progressively in all PBS immunized mice. Impressively, however, ECD/Her2 immunization fully protected 3 out of 4 (75%) mice against tumor growth (figure 7).

## Discussion

In the present study we showed that immunization with the mannosylated extracellular domain of the human Her2/neu receptor produced in yeast *P. pastoris *can induce anti-Her2/neu antibodies capable of reducing the proliferation rate of SK-BR-3 cells *in vitro*. In addition, mannosylated ECD/Her2 vaccination elicits specific CTL responses against Her2/neu overexpressing tumor cells. Both humoral and cellular responses delay and in some cases even prevent the formation of tumors in challenged mice.

The era of anti-Her2/neu vaccine development began when it was shown that a certain percentage of breast cancer patients with Her2/neu+ tumors had pre-existent T- and B-cell mediated immunity to the Her2/neu protein [4]. Since then several attempts were made towards this direction. Several studies reported that mice immunized with DNA encoding full length or truncated forms of Her2/neu developed resistance against Her2/neu - overexpressing tumors, via mechanisms involving antibodies and CD8^+ ^T cells [5,10,19]. Peptides derived mostly from cryptic sequences of the extracellular or the intracellular domain of Her2/neu, have also been used as vaccines leading to the generation of CTL-specific responses with potent antitumor immunity [6-8]. Whole fragments of Her2/neu have, finally, been used as candidate vaccines against Her2/neu overexpressing tumors. The intracellular domain of Her2/neu produced in *E. coli *has been shown to partially inhibit the growth of cancer cells [10]. However the extracallular fragment produced in various transfected cell lines such as DHFR/G8, BHK/erbB2 and L cells which confer normal mammalian glycosylation and not mannosylation, could not confer any protection against growing tumors [9,11]. In contrast, when the ECD/Her2 was fused to cytokines like GM-CSF [12], or combined with anti-Her2/neu IgG3 antibodies fused to cytokines IL-2, IL-12 and GM-SCF [11], both humoral and cell-mediated responses against Her2/neu were observed.

The soluble expression of human ECD/Her2 molecule in yeast *P. pastoris *produced a highly mannosylated recombinant protein. Mannosylated antigens are recognized by mannose receptors (MRs), found on macrophages and dendritic cells. These receptors have the capacity to direct internalized antigens into endocytic and phagocytic pathways [13]. As a result, antigens are presented in major histocompatibility complexes class I and II and subsequently CD4^+ ^T cells [14] and CD8^+ ^T cells are activated [15]. According to this, the aim of our study was to evaluate whether the mannosylation of the ECD/Her2 produced in *P. pastoris *can potentiate the anti-tumor response of vaccinated mice. By using two tumor/mouse models we could show that vaccination with the mannosylated ECD/Her2 could induce effective antitumor activity *in vivo*. In particular, the growth of D2F2/E2 (transfected to express human HER-2/neu), but not of wild-type D2F2 cells, was significantly slowed down in 7 of 9 vaccinated BALB/c animals whereas the remainders became long-term survivors. In the second model, we utilized the HHD mouse model as a platform for active antitumor immunization. The relevance of the HHD model at identifying actual HLA.A2.1-restricted peptides has been demonstrated by the ability of these peptides to stimulate *in vitro *human PBMC derived from both healthy and cancer donors [20-22]. There is evidence for the overall correlation between the HLA.A2.1-binding capacity of the peptides and their immunogenicity in HHD mice [23]. We and others have previously shown that HHD-derived T cells can lyse not only peptide-pulsed targets but also tumor cells, which express the corresponding epitope on their surface as a result of endogenous tumor-associated antigen processing [8,20-24]. HLA.A2.1 transgenic HHD mice rejected (3 of 4) the transplantable ALC tumors stable transfected to express HLA.A2.1 and human Her2/neu. Thus, the *in vivo *efficacy of the mannosylated ECD/Her2 in the models used herein suggests that in addition to the antibody recognized epitopes, HLA.A2.1-restricted epitopes exist on this domain which are immunogenic enough to induce efficient antitumor CTL responses *in vivo*. Although, this has not been formally proven herein, the data from the *in vivo *induction of CTL in BALB/c animals lysing in vitro D2E2/F2 targets, suggest that this is likely to be the case.

Negative selection in the thymus is capable of deleting almost all of self-reactive T cells [25,26]. However, because this process is not complete, several peripheral tolerance mechanisms persist [26]. Because most known tumor antigens, including Her2/neu, come from self-protein, these incompletely removed self-reactive T cells must be activated before being applied to tumor immunotherapy. Recently, several reports have shown that xenogenic vaccination could break self-tolerance and inhibit the progression of established tumor in syngeneic or transgenic mouse models [27-29]. In our study, xenogenic immunization with recombinant human ECD/Her2 induced robust antitumor responses against murine tumor cell lines overexpressing human Her2/neu. Given the high homology between human and murine Her2/neu [30], we propose that our human ECD/Her2 vaccine will also induce cross-reactive CTL against murine Her2/neu, rejecting transplantable tumors expressing murine Her2/neu, thus breaking tolerance against self-Her2/neu, as also proposed in other tumor models based on xeno-vaccinations [8,31]. In addition, given that ECD/Her2 is a potential source of immunogenic determinants recognized by CD4^+ ^T helper cells [32], then it is obvious that activation of the latter cell population will also lead to generation of B cell clones producing anti-Her2/neu antibodies, thus overcoming any tolerance mechanisms affecting the B cell compartment.

The data from the injections in BALB/c mice also suggest that in addition to the HLA.A2.1, H-2K^d ^and D^d^-restricted CTL epitopes on ECD/Her2 should exist. Whether these are the same with the HLA.A2.1-restricted ones binding with different affinities to the mouse class I alleles, or represent distinct ones, remains to be determined. Thus, the identification of the immunogenic epitopes on ECD/Her2 represents the major aim of our future studies. Once these have been identified, work will be needed to examine their immunogenicity in the human system.

## Conclusion

Taken altogether, our results show that mannosylation of ECD/Her2 increases its capacity to act as an immunogenic molecule *in vivo*, being effective as a vaccine. This may have a broader applicability as a general concept for increasing the potency of long-peptide or protein vaccines.

## Competing interests

The authors declare that they have no competing interests.

## Authors' contributions

AM, AD and CNB designed the study. AD did the experiments and drafted the manuscript. CG was involved in cell cultures and purification of ECD/Her2. All authors approved the final version of the manuscript.

## Pre-publication history

The pre-publication history for this paper can be accessed here:

http://www.biomedcentral.com/1471-2407/9/386/prepub
